# Minority status and mental distress: a comparison of group density effects

**DOI:** 10.1017/S0033291716001835

**Published:** 2016-08-15

**Authors:** P. Schofield, J. Das-Munshi, L. Bécares, C. Morgan, V. Bhavsar, M. Hotopf, S. L. Hatch

**Affiliations:** 1 Institute of Psychiatry, Psychology & Neuroscience, King’s College London, UK; 2 The University of Manchester, Manchester, UK

**Keywords:** Depression, psychosis, suicide, social determinants

## Abstract

**Background:**

It has been observed that mental disorders, such as psychosis, are more common for people in some ethnic groups in areas where their ethnic group is less common. We set out to test whether this ethnic density effect reflects minority status in general, by looking at three situations where individual characteristics differ from what is usual in a locality.

**Method:**

Using data from the South East London Community Health study (*n* = 1698) we investigated associations between minority status (defined by: ethnicity, household status and occupational social class) and risk of psychotic experiences, common mental disorders and parasuicide. We used a multilevel logistic model to examine cross-level interactions between minority status at individual and neighbourhood levels.

**Results:**

Being Black in an area where this was less common (10%) was associated with higher odds of psychotic experiences [odds ratio (OR) 1.34 95% confidence interval (CI) 1.07–1.67], and attempted suicide (OR 1.84 95% CI 1.19–2.85). Living alone where this was less usual (10% less) was associated with increased odds of psychotic experiences (OR 2.18 95% CI 0.91–5.26), while being in a disadvantaged social class where this was less usual (10% less) was associated with increased odds of attempted suicide (OR 1.33 95% CI 1.03–1.71). We found no evidence for an association with common mental disorders.

**Conclusions:**

The relationship between minority status and mental distress was most apparent when defined in terms of broad ethnic group but was also observed for individual household status and occupational social class.

## Introduction

Having a minority status, in terms of a defining social characteristic that differs from others in a locality, has been associated with an increased risk of mental illness (van Os *et al.* 2010; Zammit *et al.* 2010; Shaw *et al.* 2012). Most studies, to date, define minority status in terms of ethnicity where, as the proportion of ethnic minorities in a locality increases – and thus their minority status decreases – the risk of mental illness is reduced. This ‘ethnic density’ effect is well documented (Boydell *et al.* 2001; Kirkbride *et al.* 2007; Veling *et al.* 2008; Stafford *et al.* 2009). A number of explanations for the ethnic density effect have been proposed including: the absence of social support and social capital, greater vulnerability to discrimination, and increased negative self-perception and diminished social identity (Kirkbride *et al.* 2007; Yuan, 2007; Pickett & Wilkinson, 2008; Becares *et al.* 2009; Shaw *et al.* 2012). These are also relevant, to varying degrees, where there is a lack of fit between other examples of individual characteristics, such as family and socioeconomic status, and the neighbourhood social environment. However other types of minority status have received relatively little attention. One study in Maastricht found the effect of being single on schizophrenia risk was almost doubled for people living alone in areas where living alone was less common than average (van Os *et al.* 2000). Also a Swedish cohort study found that school children who were socioeconomically deprived were more likely to experience psychotic symptoms in later life if they went to a school where others were on average less deprived (Zammit *et al.* 2010). Ethnic density effects have also been shown for suicide and self-harm (Neeleman & Wessely, 1999; Neeleman *et al.* 2001; Termorshuizen *et al.* 2015) and also depression (Halpern & Nazroo, 2000; Propper *et al.* 2005; Pickett *et al.* 2009; Das-Munshi *et al.* 2010). However, few recent studies have looked at these outcomes for other types of group density. There is, though, some evidence for an association between suicide and being in a minority due to socioeconomic status, with higher rates shown for unemployed people living in low unemployment areas (Platt & Kreitman, 1985; Platt, 1986). Moreover, a recent study looked at depression and being in a minority due to low socioeconomic status but failed to find an association (Albor *et al.* 2014). A recent US survey also found lower rates of depression and anxiety among lesbian, gay and bisexual (LGB) respondents in states with a greater proportion of same-sex couples (Hatzenbuehler *et al.* 2011).

Typically, although, studies of group density effects have analysed large datasets of individual health records linked to area-level data. Relying on cases identified through psychiatric records alone risks diagnostic bias acting as a confounder. Where individual characteristics are at odds with their environment, it has been argued, they may be more likely to stand out and be picked up by mental health services (Wechsler & Pugh, 1967). Furthermore, given that the majority of mental disorders are untreated, it may be difficult to distinguish between patterns of help seeking behaviour and disease incidence, particularly for less severe disorders. Another problem with using routinely collected data is that it is usually not possible to determine length of residence, and distinguish between situations where people move to a particular type of neighbourhood as a result of mental ill health (‘social drift’) and those where the neighbourhood is itself a causal factor. Also, health records typically include very little information about the social characteristics of patients. One exception in recent years is ethnicity which is now routinely collected in UK health records, as it is in some other countries, and this may in part have contributed to the recent focus on ethnic density.

With this study we set out to test the hypothesis that different types of minority status are associated with increased risk of mental disorder. Our secondary aim was to compare the relative effects of different types of minority status on a range of outcomes in order to learn more about possible mechanisms behind these effects. In order to overcome the limitations of studies based on health records alone, as outlined above, we used community survey data collected as part of the South East London Community Health (SELCoH) study. This is a large-scale community psychiatric and physical morbidity survey that includes detailed information about the social characteristics of participants including length of residence (Hatch *et al.* 2011). By linking these data with census data for the local neighbourhood we were able to examine the effect of minority status, defined by: ethnic group (Black African or Black Caribbean), household status (living alone) and occupational social class (disadvantaged) on a range of mental disorders. We were able to then relate this to three outcomes: psychotic experiences, common mental disorders and parasuicide.

## Method

### Sample

We used data collected from the initial phase of SELCoH, comprising 1698 individuals in 1075 households randomly selected from the London boroughs of Lambeth and Southwark between 2008 and 2010. Both areas are ethnically diverse and include localities with widely differing socioeconomic profiles. The overall sample was similar to the 2011 UK Census in terms of the demographic profile, particularly ethnicity and socioeconomic indicators, for this area (ONS, 2012). (For a detailed comparison see Morgan *et al.* 2014.)

### Outcomes

We assessed subclinical psychotic experiences using the Psychosis Screening Questionnaire (PSQ; Bebbington & Nayani, 1995). The PSQ is structured as a set of initial probe questions followed by secondary questions. Following previous studies that looked at psychotic experience alone, we excluded question domains related to hypomania and defined psychotic experience as any positive response to secondary questions from the remaining domains (Morgan *et al.* 2009, 2014). The presence of a common mental disorder, such as depression or anxiety based disorders, was defined as a score of ⩾12 on the revised Clinical Interview Schedule (CIS-R) questionnaire (Lewis *et al.* 1992). Parasuicide was determined by self-report, in response to the survey question: ‘Have you ever made an attempt to take your life, by taking an overdose of tablets or in some other way?’

### Predictors

Minority status was derived by comparing each individual social characteristic with the prevalence of the same characteristic in the local neighbourhood, defined as the nearest census lower super output area (LSOA). This is the most detailed area level at which relevant UK census data is available and corresponds to, on average, around 1500 people. Previous studies have shown ethnic density effects to be clearest at this detailed area level (Schofield *et al.* 2011, 2016).

Ethnicity was based on self-reported identification with either Black Caribbean or Black African census-defined ethnic groups. These are the two largest minority ethnic groups in this area, in Lambeth (11.6% Black African; 9.5% Black Caribbean) and Southwark (16.4% Black African; 6.2% Black Caribbean) (ONS, 2012). However, the number of survey participants in these groups was still small (143 Caribbean, 234 African), therefore, to improve statistical power, we collapsed both groups in the main analysis to create an overall Black category. The equivalent area ethnic density measure was derived by adding the proportion of census-derived Black Caribbean and Black African residents in each LSOA.

Single household status was based on the number of people living in each surveyed household and, at area level (LSOA), the proportion of people in single households was derived from recent census (2011) data.

Occupational social class was defined using the Registrar General's social class scale, based on current occupation (Office of Population Censuses & Surveys, 1980). This was dichotomized into advantaged (professional, managerial and technical, skilled non-manual and skilled manual) and disadvantaged (partly skilled, unskilled and unemployed) groups. The following were excluded as they could not be easily assigned to either group: student, temporary sick/disabled, retired and looking after children. At area level (LSOA), occupational social class was defined using the National Statistics Socio-Economic Classification (NS-SEC) (Chandola & Jenkinson, 1999). This was split into two categories to match the individual level measure: advantaged (higher managerial, lower managerial and intermediate) and disadvantaged (lower supervisory, semi-routine, routine and never worked/unemployed). We excluded small employers and own account workers as these could not be easily classified.

We adjusted for area deprivation using the Index of Multiple Deprivation (IMD; McLennan *et al.* 2011). The IMD is derived from national data collected on seven domains (income, employment, education, health, crime, barriers to housing and services and living environment) (Department for Communities and Local Government, 2011). We also adjusted for age and gender in each analysis, with age entered in the following age groups: 16–24, 25–34, 35–44, 45–54, 55–64, and ⩾65 years to account for nonlinear effects.

### Statistical analysis

The main analysis was conducted using multilevel logistic regression to simultaneously account for effects at three levels: (1) individual respondent, (2) household (to account for survey design) and (3) neighbourhood (LSOA). Minority status was operationalized, for each characteristic in turn, as a cross-level interaction between each individual characteristic and the area level prevalence of that characteristic. The effect of minority status on each mental health outcome, derived from the regression coefficients, was expressed in terms of a 10% decrease in the area prevalence of each characteristic for respondents with that characteristic. This follows the approach of previous ethnic density studies (Das-Munshi *et al.* 2010, 2012) and allows comparison across different types of minority status. SELCoH includes non-response weights to account for age and gender discrepancies between the study sample and the overall population sampled. When incorporating weights in a multilevel model it is usually recommended to use re-scaling methods for weights at lower levels (Pfeffermann *et al.* 1998). However, as age and gender are together highly related to the study outcomes we chose not to rescale weights, but instead entered these at the individual level only, assuming equal probability of selection at higher levels (Rabe-Hesketh & Skrondal, 2006). As a sensitivity analysis we reran the analysis both with and without weights to see whether this made any difference to our overall conclusions. We carried out a further sensitivity analysis testing for the effect of possible social drift by re-running the analysis excluding all those who had moved within the past 2 years. We also re-did the analysis using disaggregated ethnic groups to see if this would make a difference. We adjusted for area-level deprivation where relevant and all results were adjusted for age and gender.

All analyses were performed using Stata version 14 (StataCorp, 2015).

### Ethical standards

Ethical approval for the survey was granted by King's College London Research Ethics Committee (CREC/07/08–152).

## Results

### Sample characteristics

Our study sample included 1698 respondents in 1067 households and 322 LSOAs. Of these respondents, all but eight completed the PSQ, all but six completed the common mental disorders (CIS-R) questionnaire, and all but 12 responded to the parasuicide question. Ethnicity was recorded for all but two and household status for all but four respondents. For occupational social class the above exclusions, outlined above, meant that 247 (14.6%) were removed. Area-level (LSOA) data was matched for all study respondents.

A substantial minority of respondents (26%) had experienced of some form of mental distress, according to the measures we looked at, with 19% having had a psychotic like experience in the past year, 23% classified as currently having a common mental disorder (a CIS-R score of ⩾12), and 8% having attempted suicide at some point in their lives (Table 1).

**Table 1.** Description of study respondents

	Ethnicity		Household status		Occupational class		
	Black	Not black	Single household	Larger household	Disadvantaged	Not disadvantaged	Overall
Age, years, mean (s.d.)	38 (16)	41 (17)	53 (18)	38 (16)	40 (16)	40 (15)	40 (17)
Gender, male (%)	40	44	40	44	38	47	44
Psychotic experiences, PSQ case (%)	26	17	24	18	24	16	19
Depression, CIS-R ⩾12 (%)	23	24	28	23	29	21	23
Parasuicide (%)	6	8	14	7	12	5	8
Missing, frequency (%)	2 (0.1)		4 (0.2)		247 (15)		–
Neighbourhood density, mean (interquartile range)^a^	22 (16)	–	18 (5)	–	32 (22)	–	
Total, frequency (%)	377 (22)	1319 (78)	212 (13)	1482 (87)	421 (29)	1030 (71)	1698 (100%)

PSQ, Psychosis Screening Questionnaire; CIS-R, Clinical Interview Schedule – Revised.

a Percentage of people with this status in a neighbourhood.

### Evidence for cross-level interactions

We found evidence for a cross-level interaction when we included group density effects for: ethnicity and psychosis symptoms [likelihood ratio test (lrtest) *p* < 0.001] and para-suicide (lrtest *p* < 0.001); single household status and psychosis (lrtest *p* = 0.01); and some (albeit weak) evidence for social class and para-suicide (lrtest *p* = 0.06). We also found a significant difference for ethnic density and depression (lrtest *p* = 0.02) although the within group effect was non-significant.

### Ethnic density and psychosis symptoms

Taking each outcome in turn (Table 2), we found a clear relationship between ethnic density and psychotic experiences. We found that being Black in an area with 10% fewer Black people was associated with a significant increase in the odds of reporting a psychotic experience [odds ratio (OR) 1.34, 95% confidence interval (CI) 1.07–1.67]. When we looked at each ethnicity subgroup we found this was apparent, although not statistically significant (*p* = 0.136), for the Black Caribbean group only, with increased odds of psychotic experience in areas where there were fewer Black Caribbean people (OR 1.99, 95% CI 0.81–4.89) (Supplementary Table S1). However, when we looked at the overall proportion of Black African and Black Caribbean people combined, the effect for Black Caribbeans was much clearer (OR 1.48, 95% CI 1.02–2.17, *p* = 0.04) (Supplementary Table S2).

**Table 2.** Minority status and mental distress

	Psychotic experiences (PSQ)		Depression (CIS-R ⩾12)		Parasuicide	
	OR (95% CI)	*p* value	OR (95% CI)	*p* value	OR (95% CI)	*p* value
Ethnic density (if Black then effect of 10% decrease in area proportion of Black people)^a^	1.34 (1.07–1.67)	0.010	1.13 (0.88–1.44)	0.327	1.84 (1.19–2.85)	0.006
Household status (if in single household then effect of 10% decrease in area proportion of people in single households)^b^	2.18 (0.91–5.26)	0.082	0.86 (0.36–2.03)	0.723	1.65 (0.49–5.53)	0.417
Social class (if disadvantaged then effect of 10% decrease in area proportion of disadvantaged)^a^	0.88 (0.73–1.05)	0.153	1.08 (0.88–1.33)	0.452	1.33 (1.03–1.71)	0.028

PSQ, Psychosis Screening Questionnaire; CIS-R, Clinical Interview Schedule – Revised; OR, Odds ratio; CI, confidence interval.

a Adjusted for age and gender.

b Adjusted for age, gender and area deprivation.

### Other group density effects on psychosis symptoms

We found some, albeit weak, evidence of an association (*p* = 0.082) when we looked at living alone in an area with 10% fewer people who live alone and psychotic experiences (OR 2.18, 95% CI 0.91–5.26). Being in a more disadvantaged social class in an area where this was less usual (10% less) failed to make a statistically significant difference to the odds of psychotic experiences (*p* = 0.153) and in fact showed a small inverse relationship (OR 0.88, 95% CI 0.73–1.05).

### Group density and other outcomes

We found no association between the above measures of minority status and odds of common mental disorder. When we looked at parasuicide we found a clear association with ethnic density (OR 1.84, 95% CI 1.19–2.85). Again when we looked at ethnicity subgroups this effect was only retained for the Black Caribbean group, and again only when we looked at the overall Black population in each area (OR 2.32, 95% CI 1.22–4.42). We found no relationship between being in a minority due to single household status and risk of parasuicide. However, being in a disadvantaged social class where this was less common was associated with greater odds of parasuicide (OR 1.33, 95% CI 1.03–1.71). Area level deprivation did not make any difference to the results for ethnic density and occupational social class and was therefore removed from the final model. For the analysis of single household status, area deprivation made a small difference and was therefore retained as a covariate.

### Sensitivity analyses

We conducted a sensitivity analyses designed to control for social drift by restricting the sample to respondents who had been at the same address for ⩾2 years, removing approximately 35% of the original sample. We found this made very little difference other than reducing the statistical significance of the estimates (Supplementary Table S3). We also conducted a sensitivity analysis to see if our results were influenced by the method we used to incorporate survey weights. The unweighted analysis made almost no difference to our overall results (see Supplementary Table S4).

## Discussion

### Summary

We found that the experience of being in a minority due to ethnicity, single household status and occupational social class was associated with some, but not all, of the mental health outcomes we looked at. We found a strong ethnic density effect on both risk of psychotic experiences and parasuicide. We also found that living alone in an area where this was less usual was associated with increased risk of psychotic experience and that being in a disadvantaged social class in an area where this was less usual was associated with greater risk of parasuicide. We found no evidence that these group density effects were associated with common mental disorders.

### Strengths and limitations

The community survey data we used gave us a unique opportunity to compare the effects of different types of minority status on a range of mental disorders in the same study. We were able to avoid the diagnostic bias that can occur when using health records alone while the richness of the data allowed us to look at a variety of experiences of minority status. This was, though, cross-sectional data only and, while we could address issues with reverse causation where people have recently moved, we are unable to say much about the housing history of our sample beyond the previous 2 years. Therefore, we cannot rule out selection bias for mental disorders lasting more than 2 years or parasuicide prior to this. We would, though, expect `social drift’ to work in the opposite direction, so that people suffering mental distress would be more likely to move into areas with a higher proportion of people in these minority groups.

A further limitation is that, despite the survey being large (*n* = 1698), we were restricted to fairly crude definitions of minority status to achieve statistical power. We were though able to investigate, to a limited extent, ethnic density effects within ethnic subgroups. Our definition of occupational social class was, by necessity, crude as we had to match classes at individual and area levels measured on different scales. Also comparison with the social class measure is hindered by the amount of missing data, 247 (15%) missing responses, whereas this was negligible for the other measures. Our measure of single household status is also, by necessity, crude. For example, it could miss those who effectively live alone but are in shared households, due to economic necessity, although when we compared gross income for individuals living alone and those in shared households we found no overall difference.

When comparing types of minority status it is also important to bear in mind that their prevalence varied widely and this may influence the strength of the reported effects. For example, the proportion of people with single household status was small (13%) compared to the proportion of Black people (22%). Also the extent to which each status varies at neighbourhood level also differs. When comparing outcomes, caution is needed as the measures used are on different temporal scales: the CIS-R question items relate to the recent past, i.e. the past month and past week, whereas the PSQ relates to the previous year, and parasuicide is assessed over a lifetime. Also the parasuicide question is potentially more open to bias, compared to the other outcome questions, as respondents may find this particularly uncomfortable to recall or disclose to others. It is, though, worth noting that a previous study, comparing these responses with national survey data, found these were almost double the national estimates (Aschan *et al.* 2013).

As with any survey sample it is difficult to rule out selection effects. For example, we were surprised that area deprivation made relatively little difference to the results but this may simply reflect this particular sample. Last, our study is restricted to a specific urban area, south east London although this has distinct advantages as the diverse nature of the area and the richness of the data collected allowed us to look at a variety of social situations at a detailed geographical level. This is particularly important in an urban area, such as London, where localities with very different socioeconomic and ethnic profiles are often in close proximity to each other.

### Comparison with previous research

Overall our results confirm what has previously been suggested from similar studies looking at different types of minority status. However, our results showing a clear ethnic density effect associated with psychotic experiences measured using the PSQ differ from a previous national survey which failed to find an effect for the Black Caribbean group (Das-Munshi *et al.* 2012). It is possible that this is because they looked at a more dispersed national sample, of lower ethnic density areas, whereas we were able to look at a specific urban area with a high concentration of this ethnic group. It is notable that studies of schizophrenia incidence using detailed local data from the UK have shown very similar results to ours (Boydell *et al.* 2001; Kirkbride *et al.* 2007, 2014; Schofield *et al.* 2011). For example, one recent study in East London found that a 1 s.d. increase in own-group ethnic density was associated with reduced psychosis incidence [relative risk (RR) 0.70, 95% CI 0.48–0.99] among Black Africans (Kirkbride *et al.* 2014).

Looking at the association with ethnic density and parasuicide our results are in line with findings from previous work on suicide in South London. Neeleman found that the relative risk of Black and minority ethnic (BME) suicide declined (RR 0.67, 95% CI 0.51–0.87) for each s.d. increase in BME density (Neeleman & Wessely, 1999). Looking further afield, a Dutch study investigated all suicides in the four largest cities in The Netherlands and found lower risk among non-Western people (RR 0.72, *p* = 0.004) in neighbourhoods with high (over 56%) *v*. low (under 37%) non-Western minority density.

The absence of a corresponding ethnic density effect on risk of common mental disorders is perhaps not surprising given previous research. A recent review found that, out of four UK studies of Black African and Caribbean populations, only one showed a positive ethnic density effect (Shaw *et al.* 2012). Our previous study did show an inverse relationship between ethnic density and depression diagnosis for Black Africans, although for Black Caribbeans this effect appeared to work in the opposite direction (Schofield *et al.* 2016). The detrimental effect of ethnic density on the health of Black Caribbean people has been previously reported in general population studies in the UK (Stafford *et al.* 2009; Bécares *et al.* 2012). However, the present study was based on primary-care health records collected in South and East London and, as we argued, results may therefore have reflected health behaviour rather than underlying disorder.

For the effect of single household status our results are very similar to the previous Maastricht study where schizophrenia risk was almost doubled in areas with below average rates of single people (RR 10.3, 95% CI 5.6–19.2) compared to areas with above average rates (RR 4.2, 95% CI 1.9–9.3) (van Os *et al.* 2000). While we looked at a subclinical measure of psychotic experiences, the fact that our results so clearly mirror previous research on schizophrenia is perhaps not surprising as a number of studies have shown the PSQ to be similarly related to risk factors for clinical psychosis (Morgan *et al.* 2009; Das-Munshi *et al.* 2012).

One study in recent years has examined the effect of being marginalized by occupational social class. Albor *et al.* looked at a large sample (4871) of mothers from the UK Millennium Cohort Study and examined the effect of socioeconomic status, based on education and occupation, on depression or anxiety (Albor *et al.* 2014). They found that people with high status in high-status neighbourhoods had lower odds of depression or anxiety compared to those with high status in low-status neighbourhoods. However, they failed to find the converse effect, as we did, instead they did not find a neighbourhood effect for those in the low-status group. Also Zammit *et al.* (2010) used a composite measure of deprivation and found the association with schizophrenia risk was reduced (*p* = 0.06) for those living in neighbourhoods where deprivation was more common. That our study failed to replicate this, in fact showing a small effect in the opposite direction, may simply reflect the fact that deprivation is conceptually distinct from social class. Our results showing a relationship between minority status due to social class and parasuicide do, however, have a parallel with a previous study of unemployment and parasuicide. Rates of parasuicide among unemployed people in Edinburgh were shown to decline, in relative terms, when the prevailing unemployment rate increased (Platt & Kreitman, 1985; Platt, 1986). It is also worth noting that a number of studies have also shown a link between being in a minority due to socioeconomic status and poor physical health and increased mortality (Yen & Kaplan, 1999; Winkleby *et al.* 2006; Albor *et al.* 2009).

### Interpretation of results

These results suggest that there is something about being different from others in the local neighbourhood that can be deleterious to mental health. They provide support for some of the hypothesized mechanisms behind the ethnic density effect; that this is not simply about being a numerical minority, but about racialized identities and the protection from discrimination that can result from living with other ethnic minorities (Becares *et al.* 2009). One concern was that by combining two distinct ethnic groups, Black Caribbean and Black African, we risked losing much of what makes these groups coherent. In fact, we found that each had a distinct mental health profile, with 31% of Black Caribbeans reporting psychotic experiences compared to 22% for Black Africans. Therefore, it is perhaps not surprising that the ethnic density effects differed between subgroups. What was particularly interesting was that this became much clearer when we looked at specific groups in relation to the broader category of Black people living locally. This suggests that it may not be proximity to one's own ethnic group but proximity to others from a visible ethnic minority that is the relevant factor. As others have argued, it seems it is not ethnicity itself that is relevant here but rather the social situation in which ethnicity is defined (Pickett & Wilkinson, 2008). This would therefore be a useful area of investigation for future research.

The strong ethnic density effect we found does not preclude other forms of minority status also being relevant, as we have shown. It is though unclear why the observed minority social class effect on parasuicide was not also seen when we looked at psychotic experiences. It is also unclear why there is no association between parasuicide and being in a minority due to single household status. However, the much smaller area variation in single household status [interquartile range (IQR) 5% for single households compared to IQR 16% for percentage of Black people] may partly explain the absence of an effect for a relatively rare outcome such as parasuicide. It may also be that living alone is simply a more transitory state compared to occupational social class and therefore less likely to be related to a lifetime measure of parasuicide.

Furthermore, these examples of minority status are related. For example, those who are isolated in terms of ethnicity may also be isolated in terms of occupational class. We did, though, adjust for ethnicity in subsequent analyses (see Supplementary Table S5) and this made very little difference to the overall results. Also the outcomes may themselves be inter-related. However, when we adjusted for other outcomes in the analysis this made little difference to the reported results (Supplementary Table S6). It is also possible that those who have become unemployed as a result of mental ill health then find themselves in a minority in the area where they live and this could act as a confounder. We therefore also adjusted for the independent effect of being unemployed (Supplementary Table S7) but again found this made no difference to the overall results.

Looking at possible theoretical explanations for these differences; the experience of living alone, where this is less usual, may lead to a greater sense of being disconnected from others and therefore be more likely associated with psychotic experiences such as paranoia. The negative effects of being in a minority due to social class may be more likely to result in a negative self-image, due to lower perceived social position, leading to a greater tendency to parasuicide rather than psychosis.

## Conclusion and study implications

This study has been able to widen the scope of research on the relationship between group density and mental distress for a range of mental disorders. Having shown a strong ethnic density effect on psychotic experiences and parasuicide we have also shown that these effects are not unique to ethnicity but can apply to other situations where people are in a minority. Further work is now needed to explore the mechanisms behind these increased risks. For example, is it a lack of social support resulting from minority status that is most important or an increased vulnerability to discrimination, as some studies have proposed? Alternatively, is it the perception of minority status/difference that is most relevant? We therefore recommend further research looking at a range of different types of minority status to better elucidate mechanisms behind the psychosocial pathways leading to mental disorders. It would also be useful to be able to investigate the timing and duration of relevant neighbourhood exposures using longitudinal designs to better elucidate possible causal mechanisms.

## Supplementary material

For supplementary material accompanying this paper visit http://dx.doi.org/10.1017/S0033291716001835. click here to view supplementary material
