# Supplementary material for: Minority status and mental distress: a comparison of group density effects
Source: Psychol Med. 2016 Aug 15;46(14):3051–9. doi: 10.1017/S0033291716001835 (PMC5080664; doi:10.1017/S0033291716001835)
Supplement: Supplementary file 1 [file S0033291716001835sup001.docx]

**Supplementary Table S1. Minority status and mental distress – effect of living in an area with fewer of the same ethnic group for different ethnic sub-groups**

|  | Sub-clinical psychosis  (PSQ case) |  | Common mental disorder (CIS-R 12+) |  | Parasuicide |  |
| --- | --- | --- | --- | --- | --- | --- |
|  | OR (95% confidence interval) | P value | OR (95% confidence interval) | P value | OR (95% confidence interval) | P value |
|  |  |  |  |  |  |  |
| Ethnic density (If Black Caribbean then effect of 10% decrease in area proportion of Black Caribbean people)^a^ | 1.99 (0.81 to 4.89) | 0.136 | 1.8 (0.63 to 5.15) | 0.276 | 4.89 (0.74 to 32.26) | 0.099 |
| Ethnic density (If Black African then effect of 10% decrease in area proportion of Black African people)^a^ | 1.15 (0.81 to 1.62) | 0.439 | 1.06 (0.74 to 1.53) | 0.753 | 1.41 (0.68 to 2.89) | 0.354 |

^a^adjusted for age and gender

**Supplementary Table S2. Minority status and mental distress – effect of living in an area with fewer Black (Caribbean and African combined) people for different ethnic sub-groups**

|  | Sub-clinical psychosis  (PSQ case) |  | Common mental disorder (CIS-R 12+) |  | Parasuicide |  |
| --- | --- | --- | --- | --- | --- | --- |
|  | OR (95% confidence interval) | P value | OR (95% confidence interval) | P value | OR (95% confidence interval) | P value |
|  |  |  |  |  |  |  |
| Ethnic density (If Black Caribbean then effect of 10% decrease in area proportion of Black people)^a^ | 1.48 (1.02 to 2.17) | 0.041 | 1.1 (0.74 to 1.63) | 0.632 | 2.32 (1.22 to 4.42) | 0.010 |
| Ethnic density (If Black African then effect of 10% decrease in area proportion of Black people)^a^ | 1.21 (0.91 to 1.6) | 0.198 | 1.07 (0.82 to 1.41) | 0.604 | 1.37 (0.78 to 2.43) | 0.276 |

^a^adjusted for age and gender

**Supplementary Table S3. Minority status and mental distress – analysis restricted to those at the same address for past 2 years or more**

|  | Psychotic experiences (PSQ) |  | Depression (CIS-R 12+) |  | Parasuicide |  |
| --- | --- | --- | --- | --- | --- | --- |
|  | OR (95% confidence interval) | P value | OR (95% confidence interval) | P value | OR (95% confidence interval) | P value |
|  |  |  |  |  |  |  |
| Ethnic density (If Black then effect of 10% decrease in area proportion of Black people)^a^ | 1.44 (1.12 to 1.84) | 0.004 | 1.22 (0.94 to 1.59) | 0.139 | 1.72 (1.09 to 2.7) | 0.019 |
| Household status (if single household then effect of 10% decrease in area proportion of single households)^b^ | 1.97 (0.74 to 5.23) | 0.173 | 0.82 (0.33 to 1.99) | 0.657 | 1.24 (0.41 to 3.78) | 0.703 |
| Social class (if disadvantaged then effect of 10% decrease in area proportion of disadvantaged)^a^ | 0.93 (0.74 to 1.15) | 0.495 | 1.03 (0.8 to 1.31) | 0.842 | 1.13 (0.87 to 1.47) | 0.364 |

^a^adjusted for age and gender ^b^adjusted for age, gender and area deprivation

**Supplementary Table S4. Minority status and mental distress – unweighted analysis**

|  | Psychotic experiences (PSQ) |  | Depression (CIS-R 12+) |  | Parasuicide |  |
| --- | --- | --- | --- | --- | --- | --- |
|  | OR (95% confidence interval) | P value | OR (95% confidence interval) | P value | OR (95% confidence interval) | P value |
|  |  |  |  |  |  |  |
| Ethnic density (If Black then effect of 10% decrease in area proportion of Black people)^a^ | 1.35 (1.09 to 1.68) | 0.006 | 1.14 (0.89 to 1.47) | 0.294 | 1.89 (1.19 to 3.01) | 0.007 |
| Household status (if single household then effect of 10% decrease in area proportion of single households)^b^ | 2.25 (0.94 to 5.39) | 0.068 | 0.85 (0.35 to 2.03) | 0.710 | 1.91 (0.53 to 6.86) | 0.320 |
| Social class (if disadvantaged then effect of 10% decrease in area proportion of disadvantaged)^a^ | 0.89 (0.74 to 1.07) | 0.215 | 1.08 (0.88 to 1.33) | 0.456 | 1.36 (1.03 to 1.78) | 0.027 |

^a^adjusted for age and gender ^b^adjusted for age, gender and area deprivation

**Supplementary Table S5. Minority status and mental distress – models adjusted for ethnicity**

|  | Psychotic experiences (PSQ) |  | Depression (CIS-R 12+) |  | Parasuicide |  |
| --- | --- | --- | --- | --- | --- | --- |
|  | OR (95% confidence interval) | P value | OR (95% confidence interval) | P value | OR (95% confidence interval) | P value |
| Household status (if in single household then effect of 10% decrease in area proportion of people in single households)^a^ | 2.19 (0.9 to 5.35) | 0.085 | 0.91 (0.38 to 2.2) | 0.842 | 1.69 (0.5 to 5.72) | 0.399 |
| Social class (if disadvantaged then effect of 10% decrease in area proportion of disadvantaged)^b^ | 0.90 (0.75 to 1.09) | 0.286 | 1.05 (0.86 to 1.29) | 0.636 | 1.28 (0.98 to 1.66) | 0.068 |

^a^adjusted for age, gender, area deprivation and ethnic group ^b^adjusted for age, gender and ethnic group

**Supplementary Table S6. Minority status and mental distress – adjusted for other mental health outcomes in the study**

|  | Psychotic experiences (PSQ) – further adjusted for parasuicide |  | Parasuicide – further adjusted for psychotic experiences |  |
| --- | --- | --- | --- | --- |
|  | OR (95% confidence interval) | P value | OR (95% confidence interval) | P value |
|  |  |  |  |  |
| Ethnic density (if Black then effect of 10% decrease in area proportion of Black people)^a^ | 1.30 (1.04 to 1.62) | 0.022 | 1.67 (1.08 to 2.59) | 0.021 |
| Household status (if in single household then effect of 10% decrease in area proportion of people in single households)^b^ | 2.12 (0.92 to 4.89) | 0.079 | 1.32 (0.41 to 4.25) | 0.637 |
| Social class (if disadvantaged then effect of 10% decrease in area proportion of disadvantaged)^a^ | 0.85 (0.71 to 1.02) | 0.076 | 1.34 (1.05 to 1.7) | 0.020 |

^a^also adjusted for age and gender ^b^also adjusted for age, gender and area deprivation

**Supplementary Table S7. Minority status and mental distress – adjusted for unemployed status**

|  | Psychotic experiences (PSQ) |  | Depression (CIS-R 12+) |  | Parasuicide |  |
| --- | --- | --- | --- | --- | --- | --- |
|  | OR (95% confidence interval) | P value | OR (95% confidence interval) | P value | OR (95% confidence interval) | P value |
|  |  |  |  |  |  |  |
| Ethnic density (if Black then effect of 10% decrease in area proportion of Black people)^a^ | 1.38 (1.1 to 1.72) | 0.005 | 1.15 (0.91 to 1.46) | 0.247 | 1.85 (1.26 to 2.74) | 0.002 |
| Household status (if in single household then effect of 10% decrease in area proportion of people in single households)^b^ | 1.98 (0.83 to 4.71) | 0.122 | 0.8 (0.34 to 1.89) | 0.609 | 1.5 (0.44 to 5.2) | 0.518 |
| Social class (if disadvantaged then effect of 10% decrease in area proportion of disadvantaged)^a^ | 0.85 (0.71 to 1.01) | 0.07 | 1.06 (0.86 to 1.31) | 0.573 | 1.26 (0.99 to 1.6) | 0.061 |

^a^adjusted for age, gender and unemployed status ^b^adjusted for age, gender, unemployed status and area deprivation
